# Nanopore sensing reveals a preferential pathway for the co-translocational unfolding of a conjugative relaxase–DNA complex

**DOI:** 10.1093/nar/gkad492

**Published:** 2023-06-02

**Authors:** Fernando Valenzuela-Gómez, Ignacio Arechaga, David Rodríguez-Larrea, Elena Cabezón

**Affiliations:** Departamento de Biología Molecular and Instituto de Biomedicina y Biotecnología de Cantabria (IBBTEC), Universidad de Cantabria- CSIC, 39011 Santander, Spain; Departamento de Biología Molecular and Instituto de Biomedicina y Biotecnología de Cantabria (IBBTEC), Universidad de Cantabria- CSIC, 39011 Santander, Spain; Biofisika Institute (UPV/EHU, CSIC), Department of Biochemistry and Molecular Biology (UPV/EHU), Leioa 48940, Spain; Departamento de Biología Molecular and Instituto de Biomedicina y Biotecnología de Cantabria (IBBTEC), Universidad de Cantabria- CSIC, 39011 Santander, Spain

## Abstract

Bacterial conjugation is the main mechanism for the dissemination of antibiotic resistance genes. A single DNA strand of the conjugative plasmid is transferred across bacterial membranes covalently bound to a large multi-domain protein, named relaxase, which must be unfolded to traverse the secretion channel. Two tyrosine residues of the relaxase (Y18 and Y26 in relaxase TrwC) play an important role in the processing of conjugative DNA. We have used nanopore technology to uncover the unfolding states that take place during translocation of the relaxase–DNA complex. We observed that the relaxase unfolding pathway depends on the tyrosine residue involved in conjugative DNA binding. Transfer of the nucleoprotein complex is faster when DNA is bound to residue Y18. This is the first time in which a protein–DNA complex that is naturally translocated through bacterial membranes has been analyzed by nanopore sensing, opening new horizons to apply this technology to study protein secretion.

## Graphical Abstract

**Graphical Abstract:**
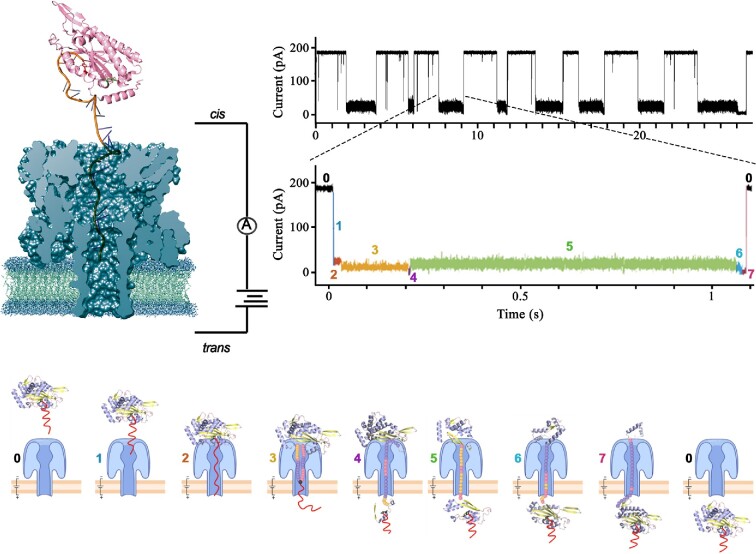

## INTRODUCTION

Antibiotic resistance has become one of the most challenging problems in health care (1,2). The main mechanism for the spread of antibiotic resistance genes is bacterial conjugation (3). In this process, a DNA strand of the conjugative plasmid is transferred bound to a pilot protein, called relaxase. Relaxases are ssDNA endonucleases that recognize a specific sequence on the plasmid (the origin of transfer) and after a cleavage reaction on the *nic* site, remain covalently bound to the 5′-end of the DNA. The resultant nucleoprotein complex is transferred through a Type IV Secretion System (T4SS), a multiprotein complex that spans the inner and outer membranes of the donor bacteria (4–6). It is important to note that conjugative relaxases are large multidomain proteins that, in some cases, like the relaxase of conjugative plasmid F, consist of >1700 amino acid residues (7). Therefore, characterization of the mechanism of relaxase transport during conjugation is not only worthwhile for its biological relevance but also for the biophysical challenge of transferring these colossal proteins across biological membranes.

R388 is one of the most well-studied and characterized conjugative plasmids. Its relaxase, TrwC, is composed of two domains. The N-terminal endonuclease domain (TrwC_R_, residues 1–293) catalyzes the cleavage and transfer of the conjugative ssDNA, whereas the C-terminal helicase domain (TrwC_H_, residues 296–966) is responsible for a 5′-3′ DNA helicase activity (8). TrwC_R_ has two catalytic tyrosines: Y18 and Y26. *In vitro*, both catalytic tyrosines are capable of bringing about the DNA cleavage reaction using oligonucleotide substrates containing the specific *nic* sequence, so the 5′-end of the cleaved DNA becomes covalently bound to the protein via Y18 or Y26 residues. However, *in vivo*, each tyrosine seems to play a distinct role. A translocation model was proposed (9), in which Y18 specifically catalyzes the initial cleavage reaction and remains covalently bound to the DNA strand that is transferred to the recipient cell. Once in the recipient cell, the protein would perform a new strand-transfer reaction to re-join both ends through Y26, leading to DNA recircularization (9,10). Given the large size of the relaxase and the internal diameter of a T4S channel—<30 Å in diameter (11–13)—protein translocation must necessarily occur in an unfolded state. In fact, recent studies have shown that translocation through a conjugative T4SS requires the unfolding of the relaxase protein (14). However, whereas several studies support the refolding of the transported protein in the recipient cell (10,15), the mechanism whereby the nucleoprotein complex is unfolded and secreted across bacterial membranes remains elusive.

The measurement of single-molecules translocating through membrane pores is the basis of nanopore technology, which has experienced a major progress in the last few years as a platform for third generation DNA sequencing (Oxford Nanopore Technologies). In addition to DNA sequencing, α-haemolysin (α-HL) nanopores are an excellent tool to study co-translocational protein unfolding at the single-molecule level. A heptameric α-HL protein forms a channel in a membrane, which allows the flow of ions in response to an imposed electrical field. A molecule translocating the nanopore produces a modulation in the current, which can be readily measured with sub-pA and sub-ms resolution. The α-HL nanopore has an upper entrance ∼24 Å wide and an internal constriction ∼14 Å wide (16). Interestingly, the electron microscopy structure of the T4SS encoded by the R388/pKM101 plasmids revealed an internal diameter of 10–20 Å (11,13,17), comparable to the internal diameter of the α-HL pore. Moreover, the forces applied in nanopore sensing are similar to those determined for the delivery of bacterial effectors (2.7–27 pN) (18,19), making α-HL nanopores a unique tool to study protein and DNA secretion.

Here, we have used nanopore technology to study the unfolding and transfer of a short ssDNA fragment of plasmid R388 covalently bound to its cognitive relaxase protein (TrwC_R_) in an attempt to answer fundamental questions about the conjugative process, such as what are the forces the relaxase–DNA complex sustains as it unfolds and crosses the channel, or what is the differential behavior of the two specific relaxase–DNA complexes. We have analyzed the co-translocational unfolding of TrwC_R_–DNA complexes with DNA either bound to Y18 (TrwC_R_(Y18)–DNA complex) or to Y26 (TrwC_R_(Y26)–DNA complex). Thus, when the ssDNA–protein complex is driven to the pore in an electric field, the ionic current signal reveals the threading of the DNA, the unfolding of the protein through distinct intermediate steps, and the translocation of the unfolded polypeptide. We have observed that both relaxase–DNA complexes undergo co-translocational unfolding, showing a distinct cyclic pattern of seven current levels separated by seven irreversible steps. The duration and intensity of each level can be associated with the barriers that the protein encounters during its passage through the nanopore. Interestingly, the TrwC_R_(Y18)–DNA complex traversed the pore 6 times faster than TrwC_R_(Y26)–DNA complex. Overall, our results reveal a particularly favorable translocation pathway that depends on a tyrosine residue that is covalently bound to DNA and open up a new avenue in the study of protein secretion by nanopore sensing.

## MATERIALS AND METHODS

### Protein expression and purification

TrwC_R_(wt) and variants TrwC_R_(Y18F) and TrwC_R_(Y26F) were expressed in *Escherichia coli* C41(DE3) strain (20). Cells were lysed in buffer 100 mM Tris–HCl pH 7.5, 300 mM NaCl, 0.001% PMSF. Lysates were collected by centrifugation at 40 000 rpm for 30 min at 4°C, diluted in buffer 100 mM Tris–HCl pH 7.5, 0.001% PMSF and applied to a P11 phosphocellulose column (Whatman). Protein was eluted in a linear gradient of NaCl and TrwC_R_-enriched fractions were pooled and applied to a HiTrap SP-HP column (GE Healthcare). After a subsequent elution in a linear gradient of NaCl, fractions were analyzed by SDS-PAGE. Protein concentration was estimated in a NanoDrop 2000c spectrophotometer (Thermo Scientific) by UV absorbance at 280 nm using an extinction coefficient of 31.5 calculated in ProtParam tool from ExPASy bioinformatics resource portal (https://web.expasy.org/protparam/). Samples were mixed with 5% (w/v) glycerol and stored at –80°C.

### Cleavage reactions and formation of TrwC_R_–DNA conjugates

Cleavage reactions were carried out by incubating TrwC_R_(wt) or TrwC_R_ variants (20 μM) with a 42-mer oligonucleotide (30 μM) containing the R388 *ni*c site (*12 + 30:* 5’-TGC GTA TTG TCT / ATA GCC CAG ATT TAA GGA TAC CAA CCC GGC-3’). The mixture was incubated at 37°C for 30 min in buffer 100 mM Tris pH 7.5, 1 mM MgCl_2_. After the cleavage reaction, TrwC_R_ remains covalently bound to the 5′-end of the resultant 30-mer oligonucleotide. Reactions were stopped by adding 10 mM EDTA and then loaded onto a HiTrap Q HP column (*GE Healthcare*) to separate free TrwC_R_ protein and DNA, from TrwC_R_–DNA conjugates. Fractions containing the complex TrwC_R_–DNA were detected by SDS-PAGE and quantified by Bradford protein assay (21). Samples were stored at –20°C with 5% (w/v) glycerol.

### α-HL pores

Nanopore technology was used to analyze the co-translocational unfolding of TrwC_R_–DNA complexes through α-HL nanopores. Wild-type monomers were expressed in an *E. coli in vitro* transcription/translation system and oligomerized into heptameric pores by incubation with rabbit red blood cell membranes (22). Then, the heptameric pores were purified by SDS-PAGE, extracted from the gel (0.2 μl, ∼1 ng/μl) and added to the *cis* compartment of a bilayer apparatus as described in (19).

### Electrical measurements and data analysis

A bilayer of 1.2-diphytanoyl-*sn*-glycero-3-phosphocholine (Avanti Polar Lipids, Alabaster, AL, USA) was made across an aperture with a diameter of 100 μm in a Teflon film (Goodfellow), which separated two compartments of 0.6 ml each, *cis* and *trans* as previously described (23). Planar lipid bilayer recordings were carried out at 22 ± 1.5 °C. The buffer was 10 mM HEPES, 2 M KCl, pH 7.2. After the insertion of a single α-HL pore from the *cis* compartment, the solution was replaced with fresh buffer by manual pipetting in order to remove excess of α-HL. Ionic currents were measured using Ag/AgCl electrodes connected to a patch-clamp amplifier (Axopatch 200B, Axon Instruments). The signal was filtered at 5 kHz (low-pass Bessel filter) and data were collected at 20 kHz with a digitizer (Digidata 1440A, Axon Instruments). In GndHCl experiments, the denaturing agent was gradually added into the *trans* compartment. Once a functional α–HL nanopore was actively translocating TrwC_R_(Y18)–DNA complexes at + 120 mV, different volumes (30, 60 or 90 μl) of GndHCl (6 M) were gently mixed with the buffer of the *trans* compartment (0.6 ml).

Raw data were first analyzed with Clampex 10.5 software (Molecular Devices) to obtain the current and dwell time of the different levels. Data organization was carried out with Microsoft Excel (Microsoft) and data analysis and plotting were performed with MATLAB R2018b (MathWorks). Dwell time distributions representing the probability density function (pdf) were fitted to a mono- or double-exponential function, according to the following formula: monoexponential pdf (*x*) = *Ak* exp(*x* – *k* exp(*x*)), where *A* is the amplitude, *k* the rate, and *x* the natural logarithm of the dwell time. Rate constants (*k*) values, expressed as s^–1^, are provided with 95% confidence interval in brackets (*n* ≈ 300). Data were collected in independent experiments (*n* > 3). The residual current of a level is defined as the ionic current of the level divided by the ionic current of the open pore. Error bars given in the text and tables represent the standard deviations (S.D.) between independent experiments (*n* = 3).

## RESULTS

### Isolation of TrwC_R_–DNA conjugative complexes

**Figure 1.**
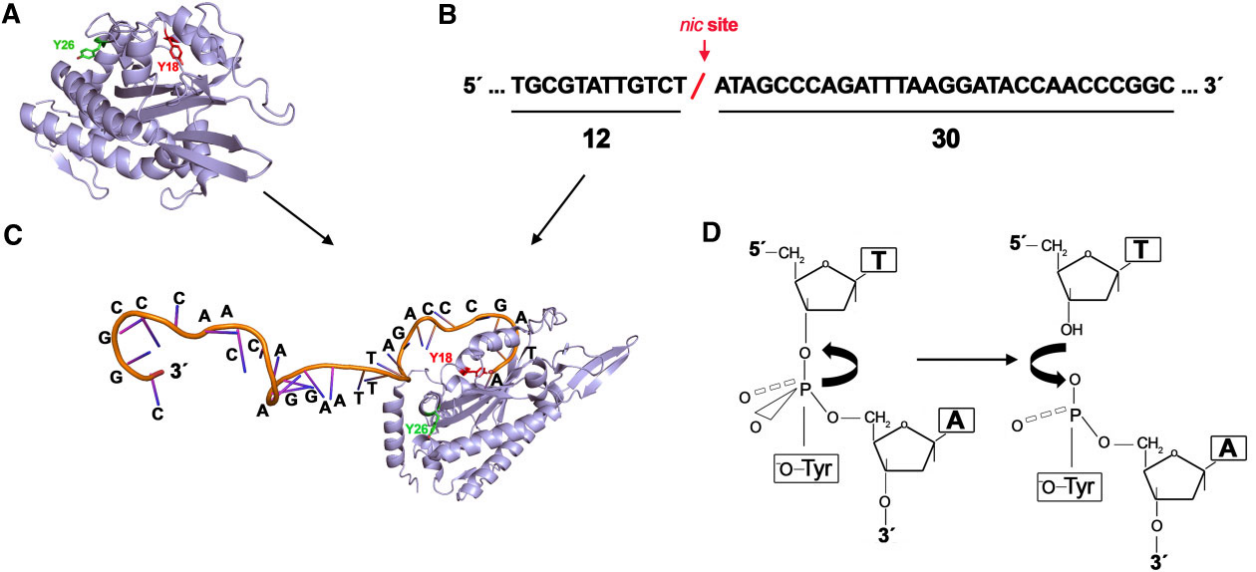
Formation of TrwC_R_–DNA covalent complexes. (**A**) TrwC_R_ structure consists of a two-layer α/β open sandwich core, also known as ‘palm’ domain (pdb: 1zm5) (44). (**B**) 42-mer oligonucleotide with the R388 plasmid sequence that contains the *nic* site for TrwC. (**C**) After the cleavage reaction, TrwC remains covalently bound to the 5′end of the resultant 30-mer sequence. (**D**) Schematic representation of the nucleophilic attack by the hydroxyl group of a catalytic tyrosine residue on the 5′-side of the DNA phosphate. This transesterification reaction results in a covalent linkage between the tyrosine residue and DNA. Adapted from (9).

The TrwC_R_ domain catalyzes conjugative DNA cleavage, which occurs via a nucleophilic attack by the hydroxyl group of a tyrosine residue on the 5′-side of the DNA phosphate. This transesterification reaction results in a covalent linkage between protein and DNA (9,24) (Figure 1).

**Figure 2.**
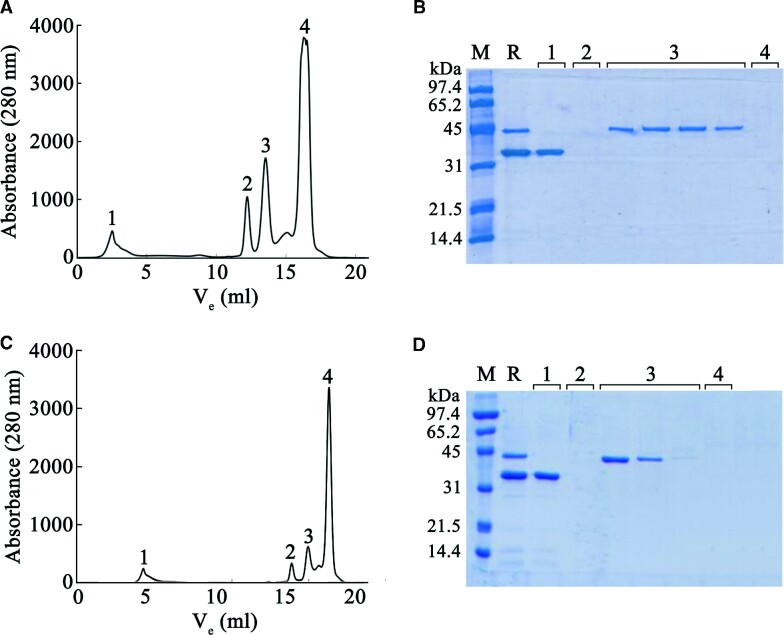
Purification and characterization of TrwC_R_–DNA conjugative complexes. In order to obtain TrwC_R_(Y18)–DNA complexes, TrwC_R_(Y26F) protein (20 μM) was incubated with a 42-mer oligonucleotide (30 μM) containing the R388 *ni*c site, and sample was loaded onto a HiTrap Q HP column (*GE Healthcare*) to separate free TrwC_R_ protein and DNA. (**A**) Elution profile. (**B**) SDS-polyacrylamide gel of the obtained fractions: (M) SDS-PAGE standards low range; (R) complex formation reaction; peaks have been numbered in order of elution (1–4). Fractions from peak 3 were harvested and used in nanopore studies. Panels (**C**) and (**D**) show the same procedure to obtain TrwC_R_(Y26)–DNA complexes except that, in this case, TrwC_R_(Y18F) protein was used to form the protein–DNA complex.

Here, we generated TrwC_R_-ssDNA complexes by incubating TrwC_R_ (20 μM) with a 42-mer oligonucleotide (30 μM) containing the *nic* sequence of plasmid R388. After the cleavage reaction, TrwC_R_ remained covalently bound to the 5′-end of the resultant 30-mer oligonucleotide (Figure 1). The reaction requires Mg^2+^ and is isoenergetic -the energy of the cleaved phosphodiester bond is stored in the form of a phosphotyrosine linkage-, which allows a subsequent ligation reaction (24). Therefore, TrwC is able to carry out cleavage and joining reactions and the resulting products represent an equilibrium between cleaved and ligated DNA. Consequently, formation of TrwC_R_–DNA complexes is not an efficient process *in vitro*, which has so far prevented the analysis of such protein–DNA complexes (9). In this work, we succeeded in stabilizing the protein–DNA complexes by the addition of EDTA. Since DNA cleavage is a magnesium-dependent reaction (25), Mg-chelation after a 30 min reaction allowed us to enhance the formation of covalent protein–DNA adducts. TrwC_R_–DNA complexes were then isolated from free protein and DNA by anion exchange chromatography (Figure 2). Fractions containing the protein–DNA complex were collected and analyzed by nanopore technology.

TrwC_R_ has two catalytic tyrosines, Y18 and Y26, which are able to form covalent protein–DNA adducts. Substitution of tyrosine with phenylalanine abolishes their catalytic activity (9). Therefore, to obtain monodisperse protein–DNA complexes, TrwC_R_(Y26F) or TrwC_R_(Y18F) variants were used to get TrwC_R_–DNA complexes with the DNA either bound to residues Y18 or Y26, respectively (TrwC_R_(Y18)–DNA or TrwC_R_(Y26)–DNA complexes).

### Translocation of a TrwC_R_(Y18)–DNA complex through an α-HL pore

**Figure 3.**
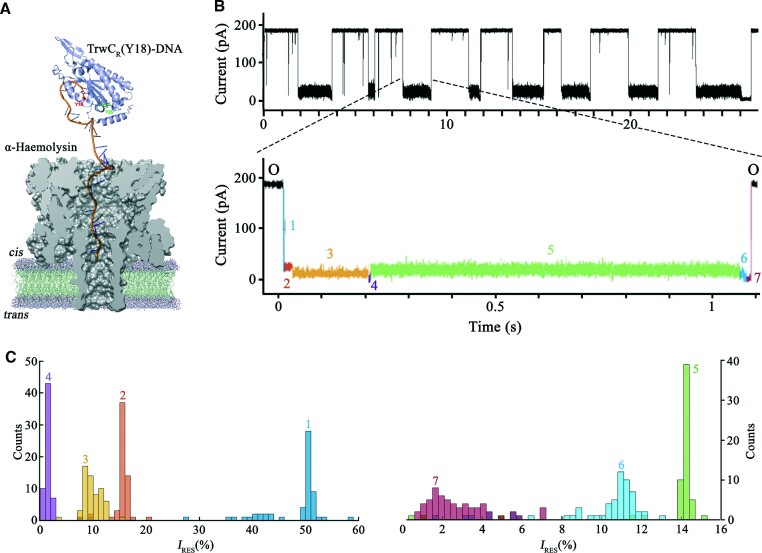
Co-translocational protein unfolding of a TrwC_R_(Y18)–DNA complex is a seven-step process. (**A**) Size-scale representation of a TrwC_R_(Y18)–DNA complex threading into the *cis* entrance of the α-HL pore. (**B**) Representative current trace of the co-translocational unfolding of a TrwC_R_(Y18)–DNA complex, obtained at +120 mV in 2 M KCl. (**C**) Event histograms of the residual current levels, expressed as *I*_RES_(%) at +120 mV, observed during the co-translocational unfolding of the TrwC_R_(Y18)–DNA complex. *I*_RES_(%) = (*I*_RES_/*I*_O_) × 100, where *I*_RES_ is the current flowing during a blockade and *I*_O_ is the current through the unblocked pore. Each level is represented with a different color code.

According to the proposed model for bacterial conjugation, a TrwC (Y18)–DNA complex would mimic the nucleoprotein substrate that is produced after DNA processing *in vivo*. Figure 3A shows a scaled representation of a TrwC_R_(Y18)–DNA complex and an α-HL nanopore used in this work (1zm5.pdb and 7ahl.pdb, respectively), highlighting the challenge of transferring a protein of 293 residues through the pore.

Membrane translocation of this conjugative complex was examined as follows. First, a single α-HL was inserted in a lipid membrane that separated two compartments (*cis* -which was at ground- and *trans*), filled with electrolyte solution (2 M KCl, 10 mM HEPES, pH 7.2). When an electrical potential of +120 mV was applied, we obtained a constant ionic current of ∼230 pA, which corresponds to the ionic current of the open pore (*I*_O_). In contrast, when we added a TrwC_R_(Y18)–DNA complex sample (1 μM), we observed a multitude of events that partially blocked the pore. More than 90% of the events showed a pattern composed of seven different ionic current levels (Figure 3B). The protein alone did not produce any significant signal and, therefore, we attribute the signal to the translocation of the complex through the nanopore by the electrophoretic capture of the ssDNA.

Data were collected and analyzed at five different voltage values, from +100 mV to +140 mV. The capture frequency depended on the applied voltage, ranging from 12 events/min at +100 mV to 55 events/min at +140 mV.

### Co-translocational unfolding of a TrwC_R_(Y18)–DNA complex is a seven-step process

Translocation of a TrwC_R_(Y18)–DNA complex through an α–HL pore showed a pattern consisting of seven ionic current levels. The characteristic signal begins with a partial blockade of the pore, step O → 1, that leads to the first observed level, with a residual ionic current of *I*_RES(%),120 mV_ = 50.5 ± 0.1% (mean ± standard deviation (S.D.), *n* = 60) (Figure 3C). *I*_RES_(%) = (*I*_RES_/*I*_O_) × 100, where *I*_RES_ is the current flowing during a blockade and *I*_O_ the current through the unblocked pore. The distribution of dwell times for level 1 (step 1 → 2) was characterized by a rate parameter *k_1→2_*_120 mV_ = 379 [315–443] s^−1^; 95% confidence interval (C.I.) in brackets; *n* = 300 (Table 1) (Supplementary Figure S1). This step was not observed in ∼11% of the events, arguably because it proceeded to the next step faster than the time resolution. Since the estimated rate of this step was 380 s^−1^ and the time resolution was about 0.2 ms, at least ∼ 8% of the events should be expected to fall below the detection limit in an exponential distribution, not being observed. The dwell time, the residual current, and the lack of voltage effect on the dwell time, are similar to previous studies that assign this step to the initial contact of the oligonucleotide with the *cis* entrance of the α-HL pore, without threading yet the transmembrane region (26).

**Table 1.** Rates of co-translocational unfolding at + 120 mV

Step	TrwC_R_(Y18)–DNA	TrwC_R_(Y26)–DNA	TrwC_R_(wt)–DNA
**1 → 2**	379 [315–443]	437 [315–559]	422 [330–514]
**2 → 3**	37 [35–39]	1051 [985–1116]	32 [27–36] / 966 [533–1399]
**3 → 4**	12 [10–15]	0.17 [0.15–0.20]	10 [8–12] / 0.19 [0.10–0.29]
**4 → 5**	275 [161–389]	155 [106–204]	244 [116–371]
**5 → 6**	0.98 [0.82–1.1]	0.96 [0.81–1.1]	0.82 [0.66–0.98]
**6 → 7**	22 [16–27]	18 [13–23]	25 [14–36]
**7 → O**	36 [25–47]	34 [27–41]	33 [21–45]

Values (s^−1^) were derived from exponential fits to dwell-time histograms, in which the data from at least three independent experiments were compiled; 95% confidence interval is provided in brackets (*n* ≈ 300).

Level 1 proceeds to a more weakly conducting level 2, of *I*_RES_(%)_,120 mV_ = 15.5 ± 0.2. Previous studies on co-translocational unfolding of artificially linked DNA–protein complexes obtained similar residual currents for the threaded oligonucleotide state, ranging from 12 to 17%, depending on the nucleotide sequence threading the pore (19). The dwell time distribution also was characterized by a rate parameter *k*_2→3120 mV_ = 37 [34–39] s^−1^ (95% C.I., *n* = 300) (Table 1). We studied the voltage dependence of this step (2 → 3) and found that the rate slightly increased with voltage, from 28 [25–30] s^−1^ at + 100 mV to 36 [31–42] s^−1^ at + 140 mV (95% C.I., *n* = 300, PCC (Pearson correlation coefficient) *r* = 0.83) (Supplementary Table S1, Supplementary Figure S2). Therefore, we attribute level 2 to a state where TrwC_R_ is placed at the top of the pore, with the oligonucleotide covalently linked through Y18 threading the pore.

Next, the signal proceeded through a series of ionic current levels (levels 3, 4, 5, 6 and 7), which we attribute to the sequential unfolding and translocation of the protein. Level 3 is of lower conductance (*I*_RES_(%),_120 mV_ = 9.5 ± 0.2) (Figure 3C). It likely corresponds to the threading of Y18 and surrounding residues into the α-HL pore, trying to move to the *trans* compartment, and trapped by the still-folded TrwC protein in the *cis* side. It is important to note that the leader DNA strand is not bound to residue 1 in the protein, but to residue 18, which means that, at pulling, residues 1–17 and residues 19-onwards will enter the nanopore all at once. In other words, two peptide segments must be threaded inside the pore at the same time, which must constitute a serious barrier to overcome. Indeed, a longer time is required to jump to the next level (the rate value of step 3 → 4 is 12 [10–15] s^−1^; 95% C.I., *n* = 300) (Table 1). Interestingly, in addition to level 2, level 3 also showed a slight voltage dependence (*k*_3→4100 mV_ = 11 [10–13] s^−1^ versus *k*_3→4140 mV_ = 16 [14–18] s^−1^; 95% C.I., *n* = 300, PCC *r* = 0.95) (Supplementary Table S1; Supplementary Figure S2). The fact that the net charge of the first protein segment is negative, as shown in a window analysis of TrwC_R_ sequence (Supplementary Figure S3), might help to thread and translocate this first peptide segment through the α-HL pore.

TrwC_R_ is too large to enter the nanopore in a folded state and, therefore, subsequent steps (level 4 onwards) must correspond to progressive protein unfolding stages. None of them showed a significant voltage dependence (Supplementary Table S1). At level 7, after completion of unfolding, the remainder of the protein traverses the pore, which opens again (step 7 → O). Although it is not possible to assign each partial unfolding step to a particular protein segment, the seven different steps described here are observed in all the analyzed events (Supplementary Figure S4) and, therefore, we can conclude that TrwC_R_ unfolding always proceeds through the same route.

### Pulling from tyrosine 26 leads to a less efficient co-translocational unfolding

**Figure 4.**
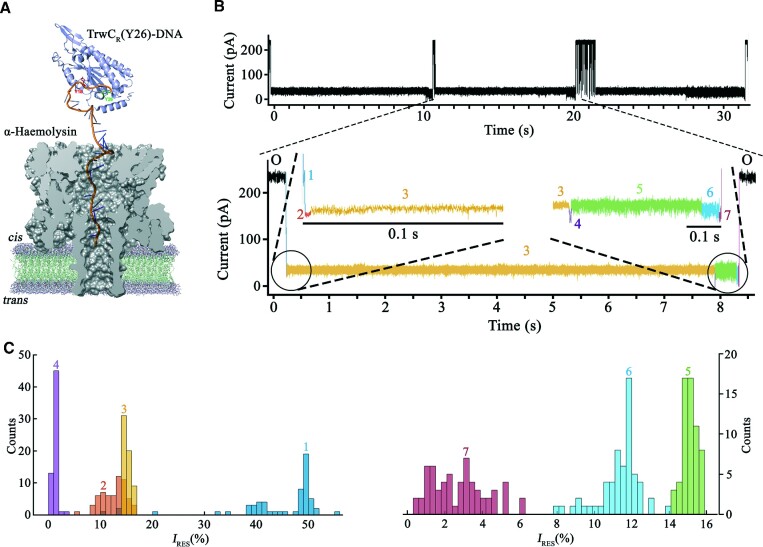
Pulling from tyrosine 26 leads to a less efficient co-translocational unfolding. (**A**) Size-scale representation of a TrwC_R_(Y26)–DNA complex threading into the *cis* entrance of the α-HL pore. (**B**) Representative current trace of the co-translocational unfolding of a TrwC_R_(Y26)–DNA complex, obtained at +120 mV in 2M KCl. (**C**) Event histograms of the residual current levels, expressed as *I*_RES_(%) at +120 mV, observed during the co-translocational unfolding of the TrwC_R_(Y26)–DNA complex. Color codes for current levels are similar to those represented in Figure 3.

Taking advantage of the catalytic activity of tyrosine 26, which also cleaves oligonucleotides containing the specific *nic* sequence, monodisperse TrwC_R_(Y26)–DNA covalent adducts were obtained by using a TrwC_R_(Y18F) mutant. The protein, covalently attached to the resultant 30-mer oligonucleotide, was pulled through the α-HL pore as described above (Figure 4A) and data were analyzed at five different voltage values, from +100 mV to +140 mV (Supplementary Table S2). Interestingly, all analyzed events also showed seven steps (Figure 4B), as previously described for a TrwC_R_(Y18)–DNA complex. Steps 4 → 5 onwards proceeded at a similar rate (Table 1, Supplementary Figures S1 and S5) and with similar *I*_RES_(%) values (Table 2). However, in steps 2 → 3 and 3 → 4, rates were significantly different from those observed for a TrwC_R_(Y18)–DNA complex (Table 1). In particular, when DNA was bound to Y26, step 2 → 3 was 28 times faster (1051 [985–1116] s^−1^ versus 37 [35–39] s^−1^, respectively, at +120 mV) (95% C.I., *n* = 300). On the contrary, step 3 → 4 was 70 times slower in this construct. This step draws particular attention, since it is rate-limiting for the translocation process (*k*_3→4,120 mV_ = 0.17 [0.15–0.2] s^−1^; 95% C.I., *n* = 300). As mentioned in the previous section, this step must correspond to the entrance of the N-terminal protein segment into the α-HL pore. The *I*_RES_(%) values for a TrwC_R_(Y26)–DNA complex at level 3 also differed significantly from those obtained for a TrwC_R_(Y18)–DNA complex (*I*_RES_ (%)_,120 mV_ = 15.01 ± 0.41 and *I*_RES_ (%)_,120 mV_ = 9.5 ± 0.2, respectively, (Table 2, Figures 3C and 4C).

**Table 2.** Residual currents of co-translocational unfolding at +120 mV

Level	TrwC_R_(Y18)–DNA	TrwC_R_(Y26)–DNA
**1**	50.5 ± 0.1	48.7 ± 0.5
**2**	15.5 ± 0.2	13.7 ± 1.6
**3**	9.5 ± 0.2	15 ± 0.4
**4**	1.3 ± 0.1	1.3 ± 0.08
**5**	14.2 ± 0.04	15.1 ± 0.3
**6**	10.8 ± 0.2	11.8 ± 0.3
**7**	2.6 ± 0.4	2.8 ± 0.6

Values (mean ± S.D.; *n* = 60) are expressed in I_RES_(%); data were compiled from independent experiments (*n* = 3).

Our results show that the co-translocational unfolding kinetics depend on the residue to which DNA is bound. TrwC_R_(Y18)–DNA and TrwC_R_(Y26)–DNA complexes proceed initially through two different routes, with distinct energy barriers to overcome, showing marked differences in their translocation kinetics. After level 3, both proceed through the same route, which is not rate limiting the translocation process. The event, as a whole, was 6 times slower in the case of the TrwC_R_(Y26)–DNA complex in comparison to a TrwC_R_(Y18)–DNA complex (median of 6.3 s versus 1 s, respectively, *n* = 300). The explanation for such an increase is the kinetic trap observed in level 3 in TrwC_R_(Y26)–DNA complexes, which reveals an intermediate that requires more than five seconds to be unfolded. *In vivo*, TrwC is thought to be secreted bound to DNA through residue Y18 (9) and, as observed here, this combination leads to a faster, and thus more efficient secretion through the nanopore.

### TrwC_R_(Y18)–DNA and TrwC_R_(Y26)–DNA complexes can be distinguished according to their translocation kinetics

**Figure 5.**
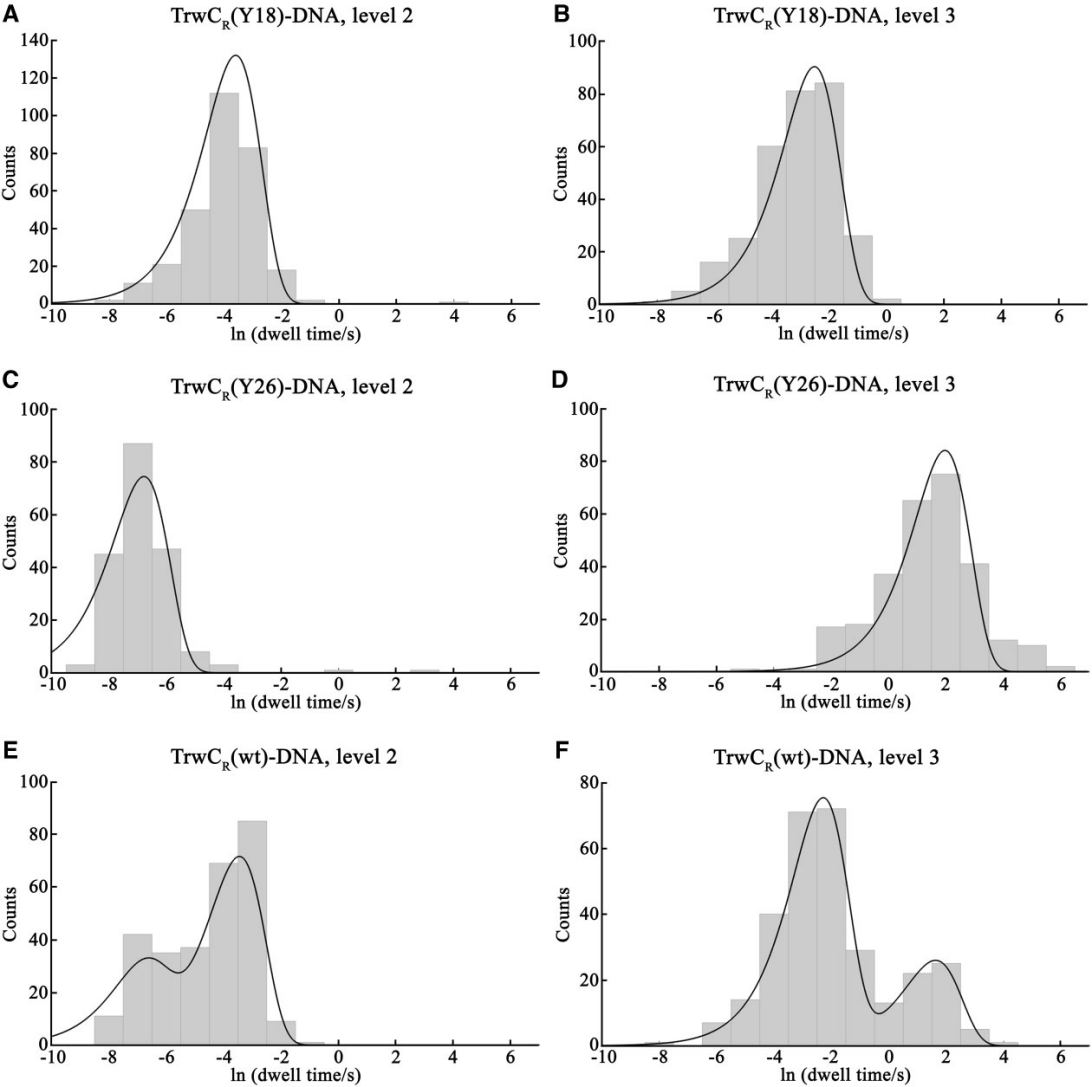
Lifetimes of levels 2 and 3 of TrwC_R_–DNA constructs during translocation. Event histograms of the dwell times in level 2 (step 2 → 3) (**A**) and level 3 (step 3 → 4) (**B**) for a TrwC_R_(Y18)–DNA complex at +120 mV. The fit is to a single exponential function and yields the rate constants *k*_2→3_= 37 s^−1^ and *k*_3→4_= 12 s^−1^ at +120 mV. Panels (**C**) and (**D**) are the event histograms of the dwell times for a TrwC_R_(Y26)–DNA complex in level 2 (step 2 → 3) (C) and level 3 (step 3 → 4) (D), respectively. Data were also fit to a single exponential function, which allowed the determination of the rate constants *k*_2→3_= 1051 s^−1^ and *k*_3→4_= 0.17 s^−1^ at +120 mV. Panels (**E**) and (**F**) are the event histograms of the dwell times for a TrwC_R_(wt)–DNA complex in level 2 (step 2 → 3) (E) and level 3 (step 3 → 4) (F), respectively. In this case, the fit is to a double exponential function and yields the rate constants *k*_2a→3_= 32 s^−1^ and *k*_2b→3_= 966 s^−1^, and *k*_3a→4_= 10 s^−1^ and *k*_3b→4_= 0.19 s^−1^ at +120 mV.

TrwC_R_ wild type protein (TrwC_R_(wt)) was incubated with the same 42-mer oligonucleotide used for previous constructs, resulting in a mixture of TrwC_R_(Y18)–DNA and TrwC_R_(Y26)–DNA complexes (9). The sample was added to the *cis* compartment of an α-HL pore, and a transmembrane potential of +120 mV was applied. All the observed co-translocational unfolding events showed seven current levels, but two different populations of molecules could be distinguished, showing different rate values in steps 2 → 3 and 3 → 4, which are the two steps shown to be different in the two types of protein–DNA complexes (Figure 5). Moreover, the dwell-time distribution in these two levels was found to be bimodal, where each peak could be assigned to a particular construct (Figure 5E, F, Table 1). The dwell-time distribution for levels 1, 4, 5, 6 and 7 did not show significant differences in TrwC_R_(Y18)–DNA, TrwC_R_(Y26)–DNA or TrwC_R_(wt)–DNA constructs (Supplementary Figures S1, S5 and S6). *I*_RES_(%) values were also very similar for levels 1, 4, 5, 6 and 7, whereas the values at levels 2 and 3 were a combination of the *I*_RES_(%) values obtained for each type of protein–DNA complex (Supplementary Figure S7), confirming the existence of two populations of molecules.

**Figure 6.**
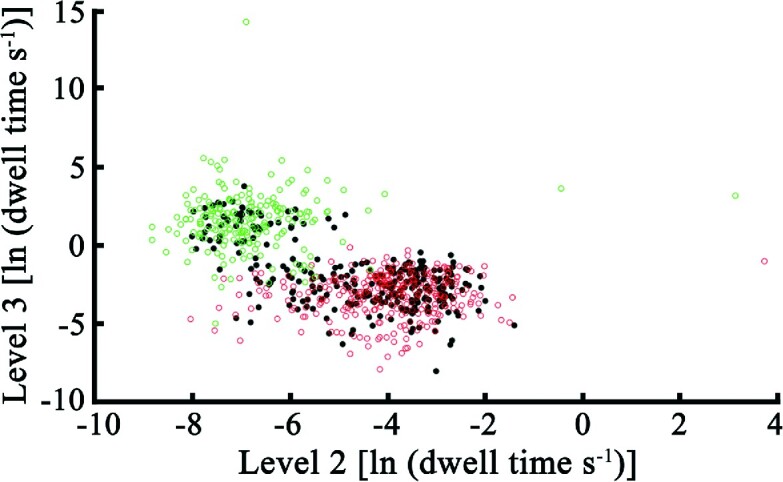
Scatter plot of the dwell time values associated to levels 2 and 3 of TrwC_R_–DNA constructs. Points represent dwell times values in levels 2 (step 2 → 3) and 3 (step 3 → 4) for TrwC_R_(Y18)–DNA (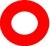), TrwC_R_(Y26)–DNA (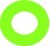), and TrwC_R_(wt)–DNA (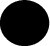) constructs, collected at +120 mV. Two differentiated populations are observed, corresponding to TrwC_R_(Y18)–DNA and TrwC_R_(Y26)–DNA samples, respectively. TrwC_R_(wt)–DNA samples show a combination of the values observed for these two populations, with a majority of events associated to a TrwC_R_(Y18)–DNA population sample. Data from at least three different experiments were used.

In a previous analysis of TrwC_R_–DNA complexes based on suicide oligonucleotides, the authors concluded that an efficient Y26 DNA cleavage only occurred after a previous Y18 DNA break (9). This means that TrwC_R_ protein molecules bound to DNA via residue Y26 would necessarily have another DNA molecule bound to Y18 (two 30-mer oligonucleotides bound to a single protein molecule). However, in the work presented here, no such double complexes were observed, since all the analyzed events showed the characteristic seven-step pattern observed either for a TrwC_R_(Y18)–DNA complex or a TrwC_R_(Y26)–DNA complex. With this analysis we can conclude that DNA cleavage through Y18 is more efficient, since TrwC_R_(Y18)–DNA complexes were more abundant (Figure 6). A *k*-means cluster analysis allowed us to assign each single event of the TrwC_R_(wt)–DNA mixture to a particular complex, either TrwC_R_(Y18)–DNA or TrwC_R_(Y26)–DNA. The results showed that 78% of the molecules corresponded to a TrwC_R_(Y18)–DNA complex, whereas only 22% had the DNA bound to residue Y26. Therefore, nanopore technology allowed us to go a step further and quantify the number of molecules of each complex, which reflects the potential of this technology to discriminate distributions within a population of molecules.

### Effect of a denaturing agent on the kinetics of the translocation process

In bacterial conjugation, once TrwC protein is translocated to the recipient cell in an unfolded state, the protein must rapidly refold to be functionally active again (10,27). We reasoned that, in T4SS, refolding of the relaxase protein in the recipient cell might act as a pulling force to complete translocation, as it occurs in T3SS (28,29). Therefore, if the unfolded translocated TrwC_R_ substrate was not able to fold back into an active conformation in the *trans* compartment of an α–HL nanopore system, an effect on the translocational pattern should be observed.

**Figure 7.**
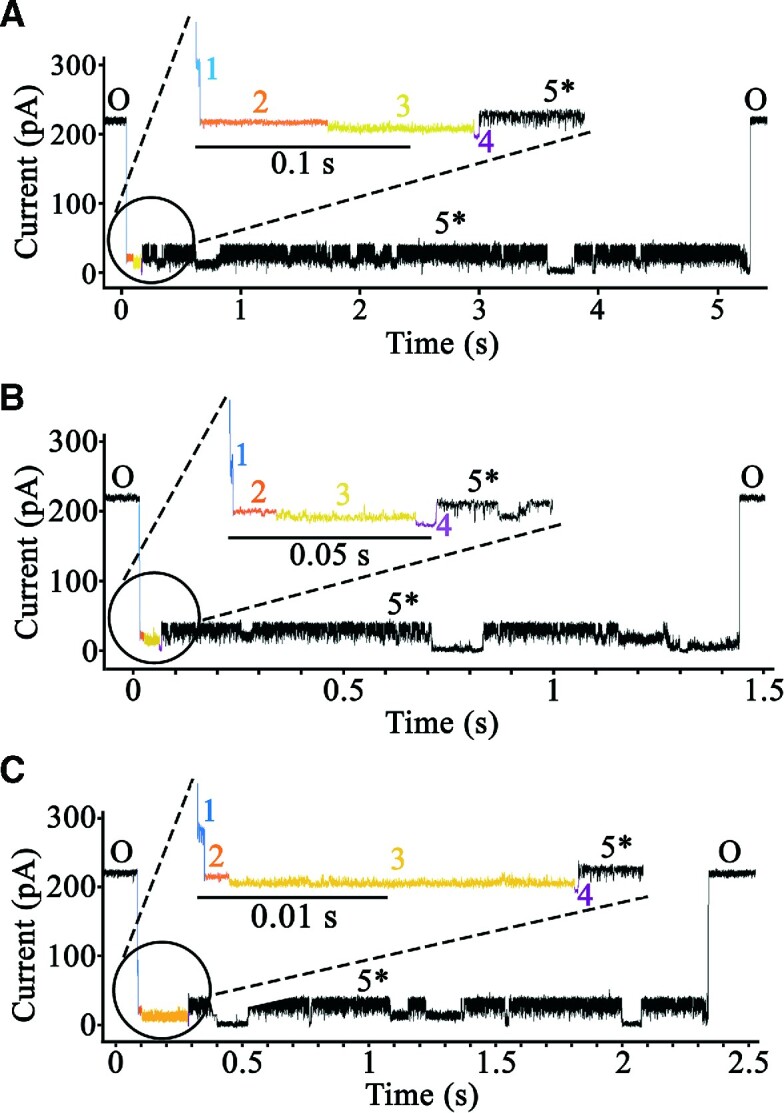
Current trace of the co-translocational unfolding of a TrwC_R_(Y18)–DNA complex in the presence of guanidinium chloride. The figure shows three different events of the co-translocational unfolding of a TrwC_R_(Y18)–DNA complex (panels **A**, **B** and **C**), obtained at +120 mV in 2M KCl, after the addition of GdnHCl (0.9 M) at the *trans* compartment of the α-HL nanopore. Levels 1 to 4 of the current trace were similar to those obtained in the absence of GdnHCl (the same color code for each level was applied). The events lacked the characteristic ionic current level 5 and, instead, from level four onwards, different current blockages without a reproducible pattern were observed until the complex was finally translocated.

In order to carry out this experiment, once a functional α–HL nanopore was actively translocating TrwC_R_(Y18)–DNA complexes, the denaturing agent guanidinium chloride (GdnHCl) was added gradually into the *trans* compartment, as described in Materials and Methods, preventing the correct refolding of TrwC at this compartment. We determined whether this had an effect on the characteristic co-translocational unfolding pattern of seven steps. After the addition of 0.9 M GdnHCl, all the translocated TrwC_R_(Y18)–DNA complexes showed a different co-translocational unfolding pattern (Figure 7). Whereas levels 1 to 4 of the current trace remained unchanged (Supplementary Figure S8), all the events lacked the characteristic ionic current levels 5, 6 and 7. Instead, from level four onwards, different current blockages with variable dwell times and *I*_RES_(%) values, and without a reproducible pattern, were observed until the complex was finally translocated (Figure 7). In addition, the median duration of the translocation process was found to be ∼2 times longer in presence of GdnHCl (1.93 versus 0.99 s in absence of GdnHCl, *n* = 106). These rates allow us to conclude that TrwC_R_(Y18)–DNA complexes are less efficiently translocated in the presence of the denaturing agent. It is important to note that the amount of GdnHCl added to the *trans* compartment (0.9 M) would not totally prevent TrwC_R_ refolding since, at this concentration of denaturant, the protein is expected to be still partially folded. Although susceptibility to unfolding by GdnHCl varies from protein to protein, higher concentrations of GdnHCl (up to 6 M) are normally required for complete protein unfolding (30).

## DISCUSSION

Bacteria can evolve rapidly by acquiring new traits through horizontal gene transfer, such as virulence, metabolic properties, and most importantly, antimicrobial resistance. Bacterial conjugation represents a major form of horizontal gene transfer and is one of the main mechanisms whereby bacteria become resistant to antibiotics (3). Plasmid DNA is transferred from a donor to a recipient cell while covalently bound to a protein (a relaxase). The transfer of this nucleoprotein complex across biological membranes is mediated by a T4SS. The internal diameter of this secretion channel is only 10–20 Å (11,13,17), which implies all proteins that transverse the channel must be unfolded for passage. According to this, it has been shown that the relaxase protein of plasmid R388, named TrwC, must be unfolded to be translocated through a conjugative T4SS (14) and refolded after its transport, since the protein is active once in the recipient cell (10). However, little is known about the mechanism behind this process. In this work, we have used nanopore technology to analyze the co-translocational unfolding of the relaxase domain of the conjugative protein TrwC (TrwC_R_) covalently bound to a 30-mer oligonucleotide. This protein–DNA complex is the result of the cleavage by TrwC_R_ of a longer oligonucleotide containing the R388 sequence at the origin of transfer. Therefore, this complex mimics the nucleoprotein substrate that is transferred in bacterial conjugation. It is important to note that TrwC_R_ domain is sufficient to carry out cleavage and transesterification reactions and, in fact, some mobilizable plasmids code for a functional relaxase protein that only consists of this domain (all relaxases belonging to class MOB_C_) (31).

TrwC_R_ has two catalytic tyrosines, Y18 and Y26. *In vivo*, each tyrosine seems to play distinct roles. It was proposed that DNA is transferred to the recipient cell covalently bound to Y18 residue (9). Once in the recipient cell, Y26 would perform a new strand-transfer reaction to re-join both ends, leading to DNA recircularization. In order to test this model, we analyzed the co-translocational unfolding of TrwC_R_–DNA complexes with DNA either bound to Y18 (TrwC_R_(Y18)–DNA complex) or to Y26 (TrwC_R_(Y26)–DNA complex. When translocated through α–HL nanopores, both types of constructs showed seven co-translocational unfolding steps, consisting of subsequent translocation intermediates at various stages of unfolding. The number of steps observed and, therefore, the complexity of the process seems to be linked to the size and the nature of the protein fold. Thus, the artificially DNA-tagged thioredoxin protein (ThrX), with 107 residues, showed only four steps (19). It is important to emphasize that the relaxase–DNA covalent complex analyzed in this work is a biological substrate transferred in bacterial conjugation and, in contrast to other proteins analyzed by nanopores so far (23,32), does not require any artificial tag to traverse the pore. Moreover, TrwC_R_ has 293 residues, almost three times as many as the ThrX protein. To our knowledge, this is the largest protein translocated through an α-HL nanopore. In addition, it does not require additional proteins, such as the bacterial unfoldase ClpX at the *trans* compartment, used in other studies to facilitate the transport by capturing the protein at the exit of the nanopore (33).

**Figure 8.**
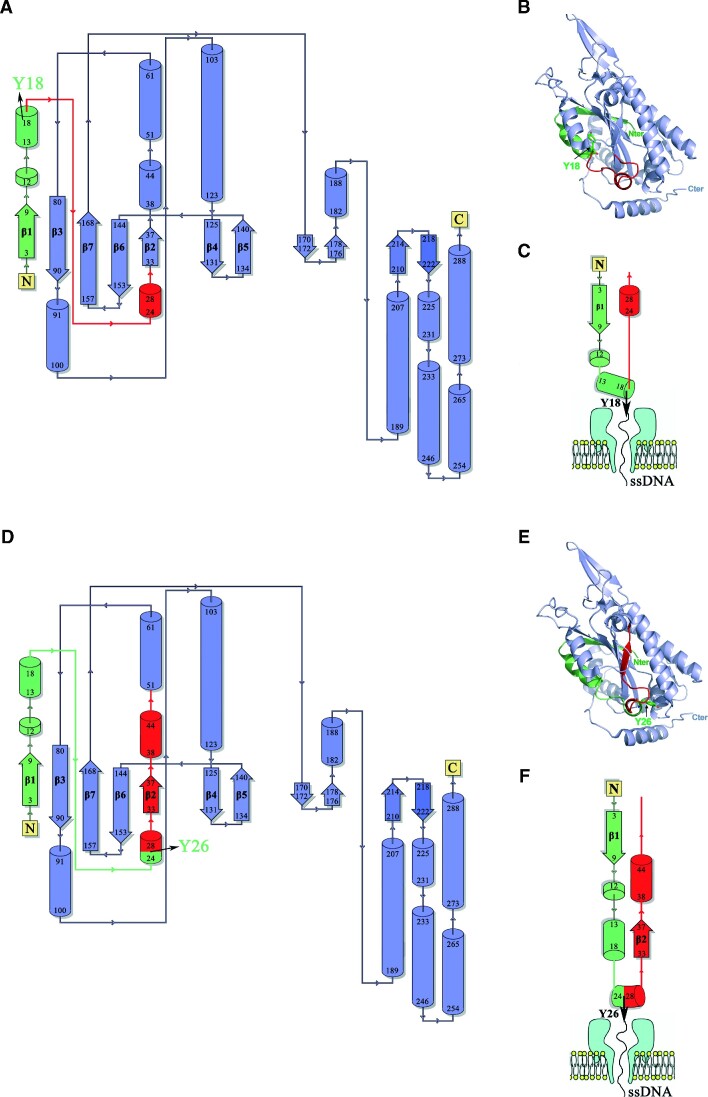
Structural barriers to overcome in TrwC_R_ unfolding. The figure represents the 3D structure of TrwC_R_ (pdb: 1zm5) (43) and its topological diagram. When TrwC_R_ is pulled from residue Y18, two peptide segments must be threaded inside the nanopore: 17 amino acids that stretch from the N-terminus to Y18 (in green), and an equivalent segment from residue Y18 onwards (in red) (panels **A**–**C**). When TrwC_R_ is pulled from residue Y26, the number of residues from each segment that enter the nanopore is higher. There will be 25 residues from the N-terminus (in green) plus residues 27-onwards on the other side (in red). In this latter case, the second segment includes the β2-strand and, therefore, this first unfolding event involves the disruption of the antiparallel five-stranded β-sheet (panels **D**–**F**). Arrows correspond to β-strands while helices are represented as cylinders.

Analyzing the co-translocational unfolding pattern, the duration and intensity of each step can be associated with a particular structural arrangement. Therefore, an inspection of the three-dimensional structure of TrwC (Figure 8, Supplementary Figure S9) allowed us to describe, for each level, the obstacles that the protein is likely to overcome as it crosses the nanopore. Level 1 must correspond to the initial contact of the oligonucleotide with the *cis* entrance of the α-HL (26). Thus, we attribute level 2 to a state where TrwC_R_ is placed at the top of the pore, with the oligonucleotide covalently linked through Y18 threading into the α–HL pore, as previously described for the ThrX protein (19), where similar residual currents for the threaded oligonucleotide state were observed. Accordingly, the results show that this level (step 2 → 3) exhibited a slight increase in the rates at higher voltage values, due to the electronegative charge of the DNA. Level 3, of lower conductance, likely corresponds to TrwC_R_ entrance into the α-HL pore. Since the leader DNA is not bound to residue 1 in the protein, but to either residue 18 in a TrwC_R_(Y18)–DNA complex or residue 26 in a TrwC_R_(Y26)–DNA complex, the barrier to overcome at this stage must be different and, in fact, this is what we observed. In a TrwC_R_(Y26)–DNA complex, level 3 was 70-fold slower than in a TrwC_R_(Y18)–DNA complex. The duration of this step draws particular attention, since it is the longest of the entire translocation process, meaning that the protein needs more than 4 s to overcome this barrier (step 3 → 4), in comparison to the 75 ms required when DNA is bound through residue Y18. This kinetic trap is clearly rate-limiting for the translocation process of TrwC_R_(Y26)–DNA complex.

TrwC_R_ structure presents two domains. The first domain comprises residues 1–168 and is a two-layer α/β sandwich domain, also known as ‘palm’ domain, consisting of an antiparallel five-stranded β-sheet with two helices flanking one face of the sheet (24). When TrwC is pulled from Y18, residues 1–17 and residues 19-onwards will enter the nanopore at the same time. Residues 1–17 can be released by dismantling first a short helix (residues 11–17) and then unzipping the β1-strand (residues 1–9), whereas amino acids on the other side of the DNA attachment site (residues 19–33) probably do not offer so much resistance, since they are part of an unstructured region, poorly resolved in the crystal structure (Figure 8A). By contrast, the number of residues that enter the nanopore from both sides of the attachment site increase in a TrwC_R_(Y26)–DNA complex. There are 25 residues from the N-terminus plus residues 27-onwards on the other side (Figure 8d). Moreover, residues 33–37 are part of the β2-strand that has to be unzipped from the other edge of the central β-sheet, which would offer higher mechanical resistance to unfolding in comparison with a TrwC_R_(Y18)–DNA complex. Such a change in the structure near the point where the force is applied (either residue Y18 or Y26) would explain the long unfolding time observed in level 3 for the TrwC_R_(Y26)–DNA complex.

Level 4 presents the lowest conductance of all the steps in the translocation process but, comparatively, it resolves very rapidly. It might correspond to the co-translocational unfolding of the two α-helices that connect β2 and β3 strands (residues 38–61) and the next destructured loop (residues 62–79), which would diffuse easily through the pore before encountering the central β-sheet. On the contrary, step (5 → 6) presents the lowest rate (Table 1) and it likely corresponds to the unfolding of such a central core (β3–β7 strands, residues 80–168) (Figure 8, Supplementary Figure S9). Therefore, in TrwC_R_(Y18)–DNA complexes, level 5 is the level that requires more time in the translocation process. Levels 6 and 7 would correspond to the unfolding of the second domain: the helical C-terminal domain known as ‘fingers’ (24). At level 7, after completion of unfolding, the remaining segment of the protein traverses the pore, which is then unblocked (step 7 → O). The seven different levels described here are observed in all the analyzed events (from level 4 onwards, rates and *I*_RES_(%) values were similar in both constructs) and, therefore, allow us to conclude that TrwC_R_ unfolding always proceeds through the same route. Moreover, we can also conclude that a TrwC_R_(Y18)–DNA complex is more efficiently translocated through the pore (each event occurs in less than 1 s, in comparison with the 6 s on average required for TrwC_R_(Y26)–DNA translocation). The success in transferring the genetic material depends, to a great extent, on how rapidly the process occurs, since maintaining close contacts between donor and recipient cells is a limiting factor in some environments. Thus, this result would support the idea that DNA is translocated to the recipient cell covalently bound to residue Y18. The translocation rate observed in our experiments is comparable to that of Type III secretion systems (T3SS), used by bacteria to deliver virulence proteins into the cytosol of host cells also in an unfolded state (29). In *Salmonella*, for instance, the rate of protein secretion has been measured at 7 to 60 proteins per cell per second (34). Bacterial conjugation, however, demands more time since the process requires the transfer of a complete plasmid (35).

Within this work, we also demonstrate the potential of nanopore technology to distinguish and classify different protein–DNA complexes in a bulk solution, since each molecule shows a particular and consistent pattern. When TrwC_R_(wt) is incubated with the same oligonucleotide used for the previous constructs, a mixture of TrwC_R_(Y18)–DNA and TrwC_R_(Y26)–DNA complexes is obtained. After applying this sample to an α-HL nanopore, the same seven current levels were observed, but two different populations of molecules could be distinguished, showing different *I*_RES_(%) values in levels 2 and 3. These differences were similar to those observed for TrwC_R_(Y18)–DNA and TrwC_R_(Y26)–DNA complexes. Moreover, the dwell-time distribution in each of these two levels was found to be bimodal, where each peak could be assigned to a particular construct. Therefore, each single event of the TrwC_R_(wt)–DNA mixture could be assigned to a particular complex. A *k*-means cluster analysis allowed us to conclude that 78% of the molecules corresponded to a TrwC_R_(Y18)–DNA complex, which supports the idea that Y18 is more effective than Y26 in the first cleavage reaction.

Other relevant questions, such as how the system is energized or the complex translocated into the recipient cell, remain unclear. In T3SS, an hexameric ATPase at the base of the channel seems to be involved in the unfolding process (36). It was suggested that this ATPase could also provide energy for pushing the substrate through the secretion channel, driving protein export. T4SS present hexameric ATPases at the base of the secretion channel that might also provide energy for the initial protein–DNA pumping (37–39). Nevertheless, as it occurs in T3SSs, there is a large distance between the ATPases and the host membrane -at least 30 nm (40)- and this primary energy supply would be inefficient for the transfer of the protein–DNA complex (41). In that sense, in T3SS, it has been reported that folding of the secreted substrate upon exit from the secretion channel provides energy for pulling protein effectors through the apparatus (28,29). Therefore, another important aspect to be considered in powering the transport of the nucleoprotein complex in bacterial conjugation, is the contribution of the relaxase unfolding-refolding process. TrwC must be refolded in the recipient cell in order to recircularize the ssDNA plasmid strand. Thus, a rapid refolding of TrwC might also play an important role as a pulling force to complete the translocation process. In our nanopore system, we reasoned that if TrwC_R_ refolding was playing such a role, we should observe a different co-translocational unfolding pattern in the presence of a denaturing agent. After the addition of 0.9 M GdnHCl to the *trans* compartment, levels 1 to 4 of the current trace remained unchanged but, in subsequent steps, different current blockages without a reproducible pattern were observed until the complex was finally translocated. Such an unpredictable pattern might reflect TrwC_R_ movement attempting to advance along the pore. On average, the translocation process was faster in the absence of GdnHCl (median duration of the translocation process of 1.93 s versus 0.99 s). Therefore, our results are compatible with the idea that protein refolding after secretion helps to pull the complex through the pore, which is in agreement with biophysical studies that show that upon denaturation of the relaxase by temperature or chaotropes the protein is able to recover the native state without the assistance of external agents (15).

In summary, this is the first time that the transfer across membranes of a conjugative complex has been studied at a single molecule level, uncovering the different partly unfolded states that take place during translocation. Other important questions, such as the energy source required for translocation in the cellular process, remain unsolved. It is tempting to speculate that intrinsic structural properties in TrwC that allow rapid refolding have been selected by evolutionary pressure, favouring a particular unfolding and translocation pathway.

## Supplementary Material

gkad492_Supplemental_File

## Data Availability

The authors confirm that the data supporting the findings of this study are available within the article and its supplementary materials.
