# Correction to ‘Nanopore sensing reveals a preferential pathway for the co-translocational unfolding of a conjugative relaxase–DNA complex’

**DOI:** 10.1093/nar/gkad1226

**Published:** 2023-12-18

*Nucleic Acids Research*, Volume 51, Issue 13, 21 July 2023, Pages 6857–6869, https://doi.org/10.1093/nar/gkad492

The authors wish to introduce the following corrections to their article.

- David Rodríguez-Larrea has been added to the author by-line as joint corresponding author with Dr Cabezón:

Fernando Valenzuela-Gómez^1^, Ignacio Arechaga^1^, David Rodríguez-Larrea^*,2^ and Elena Cabezón^*,1^

- Affiliations have been updated to:

^1^Departamento de Biología Molecular and Instituto de Biomedicina y Biotecnología de Cantabria (IBBTEC), Universidad de Cantabria- CSIC, 39011 Santander, Spain

^2^Biofisika Institute (UPV/EHU, CSIC), Department of Biochemistry and Molecular Biology (UPV/EHU), Leioa 48940, Spain

- Corresponding authors details have been updated to:

* To whom correspondence should be addressed. Tel: +34 942202033; Email: cabezone@unican.es. Correspondence may also be addressed to David Rodríguez-Larrea, Email: david.rodriguezl@ehu.es.

- Funding has been updated to:

Spanish Ministerio de Ciencia e Innovación Grants [PID2019-104251GB-I00 to E.C., PID2020-119210RB-I00 to D.R.L.]; MINECO Grant [BIO2017-88946-R to D.R.L.]; Ramon y Cajal Fellowship [RYC-2013-12799 to D.R.L.]. Funding for open access charge: Spanish Ministerio de Ciencia e Innovación Grant [PID2019-104251GB-I00].

These changes do not affect the results, discussion and conclusions presented in the article. The published article has been updated.

