# Supplementary material for: Nanopore sensing reveals a preferential pathway for the co-translocational unfolding of a conjugative relaxase–DNA complex
Source: Nucleic Acids Res. 2023 Jun 2;51(13):6857–69. doi: 10.1093/nar/gkad492 (PMC10359608; doi:10.1093/nar/gkad492)
Supplement: gkad492_Supplemental_File [file gkad492_supplemental_file.pdf]

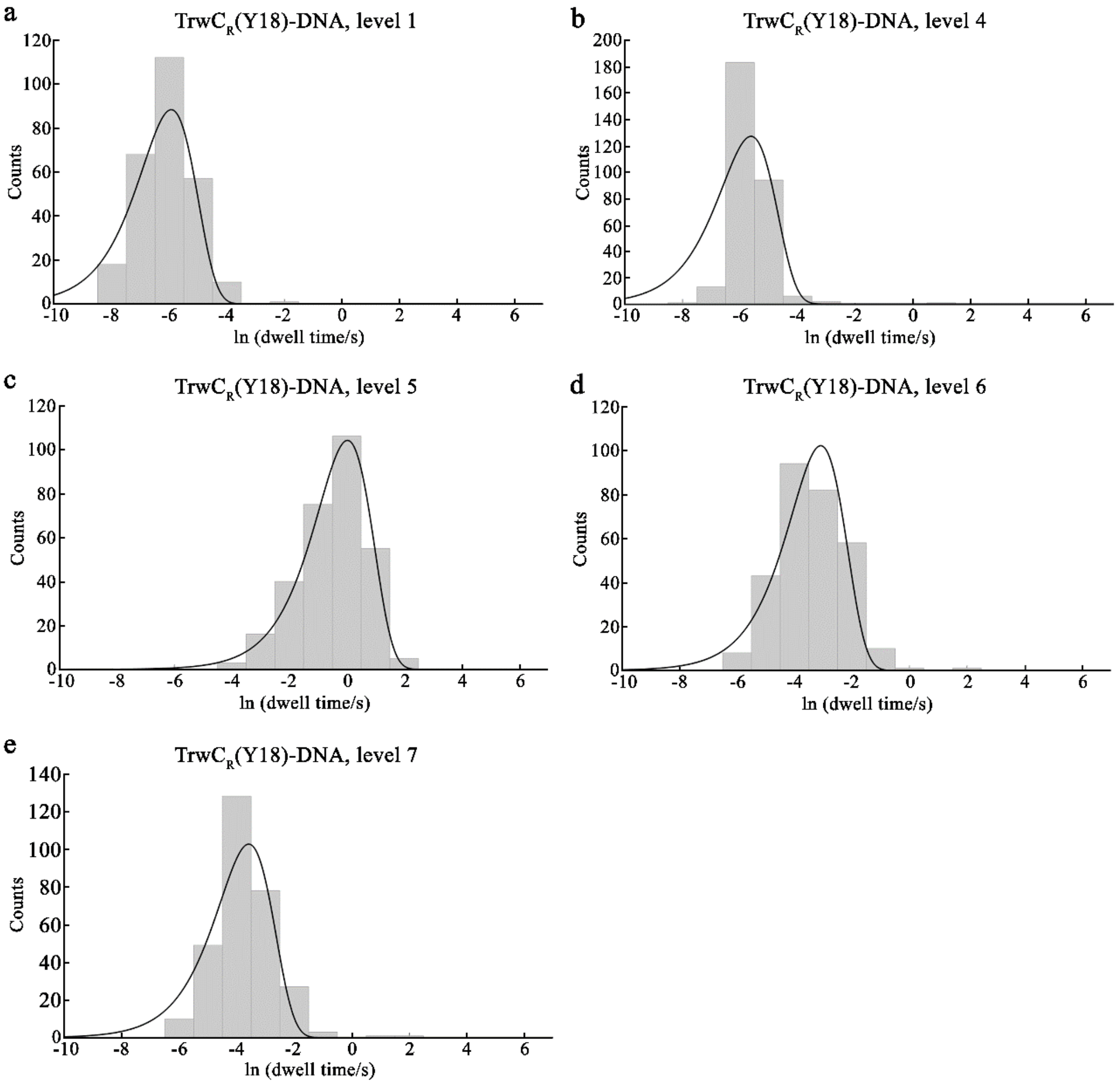

Figure S1. Dwell time distributions of a TrwC$_R$(Y18)-DNA complex during translocation. Histograms of dwell times in levels 1, 4, 5, 6 and 7 obtained at +120 mV are shown in panels a, b, c, d, and e, respectively. Levels 2 and 3 are shown in Figure 5 of the main text. Rate constant values are included in Table 1.

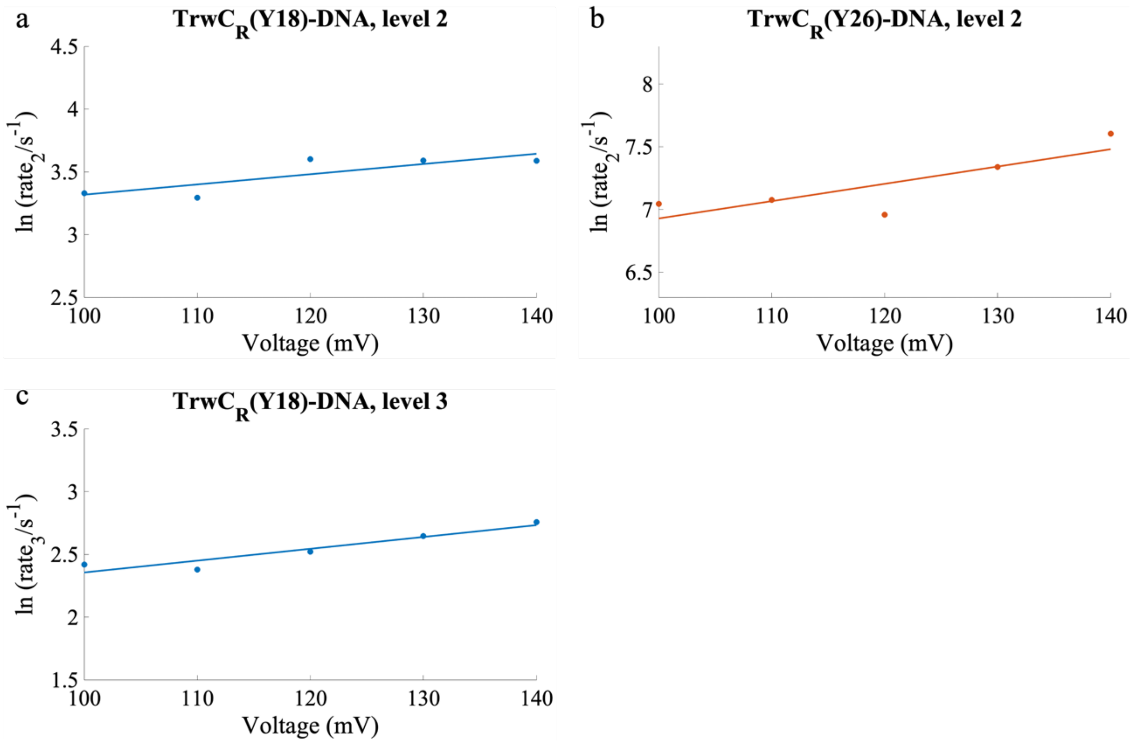

Figure S2. Voltage-dependent levels during the co-translocational unfolding process. Dwell time rates were obtained at four different electrical potentials (from +100 to +140 mV) to analyze voltage-dependence. (●) TrwC$_R$(Y18)-DNA complex. (●) TrwC$_R$(Y26)-DNA complex. Note the natural logarithm scale on the left axis. Rate values with 95 % C.I. are given in Supplementary Tables 1 and 2. Only levels that show a voltage dependence are represented in this figure.

1 59
MLSHMVLTRQDIGRAASYYEDGADDYYAKDGDASEWQGKGAEELGLSGEVDSKRFRELL
118
AGNIGEGHRIMRSATRQDSKERIGLDLTFSAPKSVSLQALVAGDAIIKAHDRAVARTLE
177
QAEARAQARQKIQGKTRIETTNLVIGKFRHETSRERDPQLHTHAVILNMTKRSDGQWRA
236
LKNDEIVKATRYLGAVYNAELAHELQKLGYQLYGKDGNFDLAHIDRQQIEGFSKRTEQI
288
AEWYAARGDPNSVSLEQKQAAKVLSRAKKTSVDREALRAEWQAAKELGIDFS

Figure S3. Window analysis of TrwC$_R$ sequence. The local charge of the polypeptide was calculated by assigning to the amino acid the charge of a stretch of 15 amino acids surrounding it. At the beginning of the translocation the net charge is negative (red), and then the sequence exhibits segments of neutral, positive (blue) and negative local net charge. The magnitude of the charge on any stretch of amino acids is low (≤ 5). The net charge on the entire polypeptide is +2.

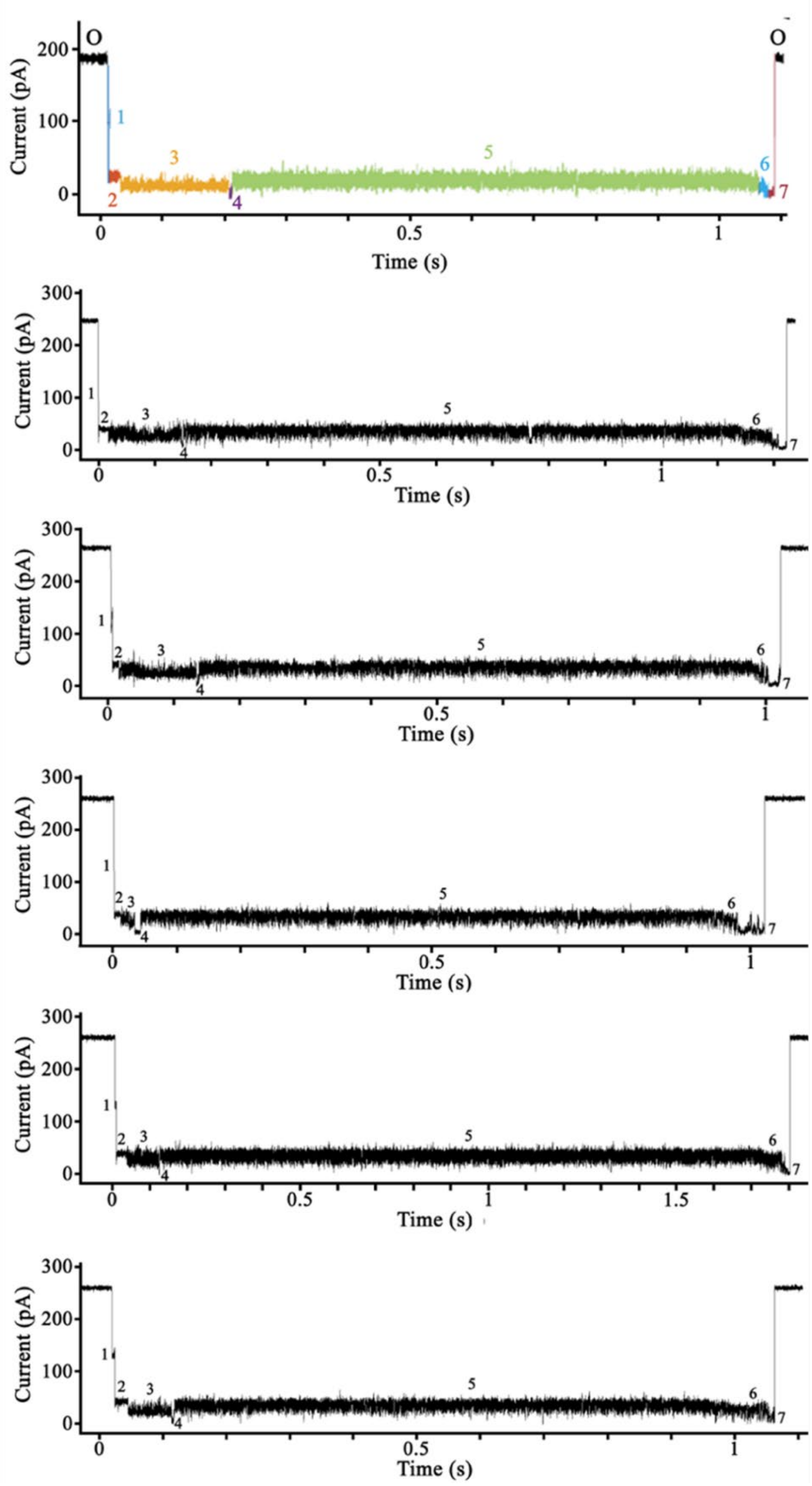

Figure S4. Co-translocational unfolding of TrwC$_R$(Y18)-DNA through an α-HL pore. The figure shows eight different events randomly selected from the collected signals. All the events comprised a repetitive pattern consisting of seven ionic current levels.

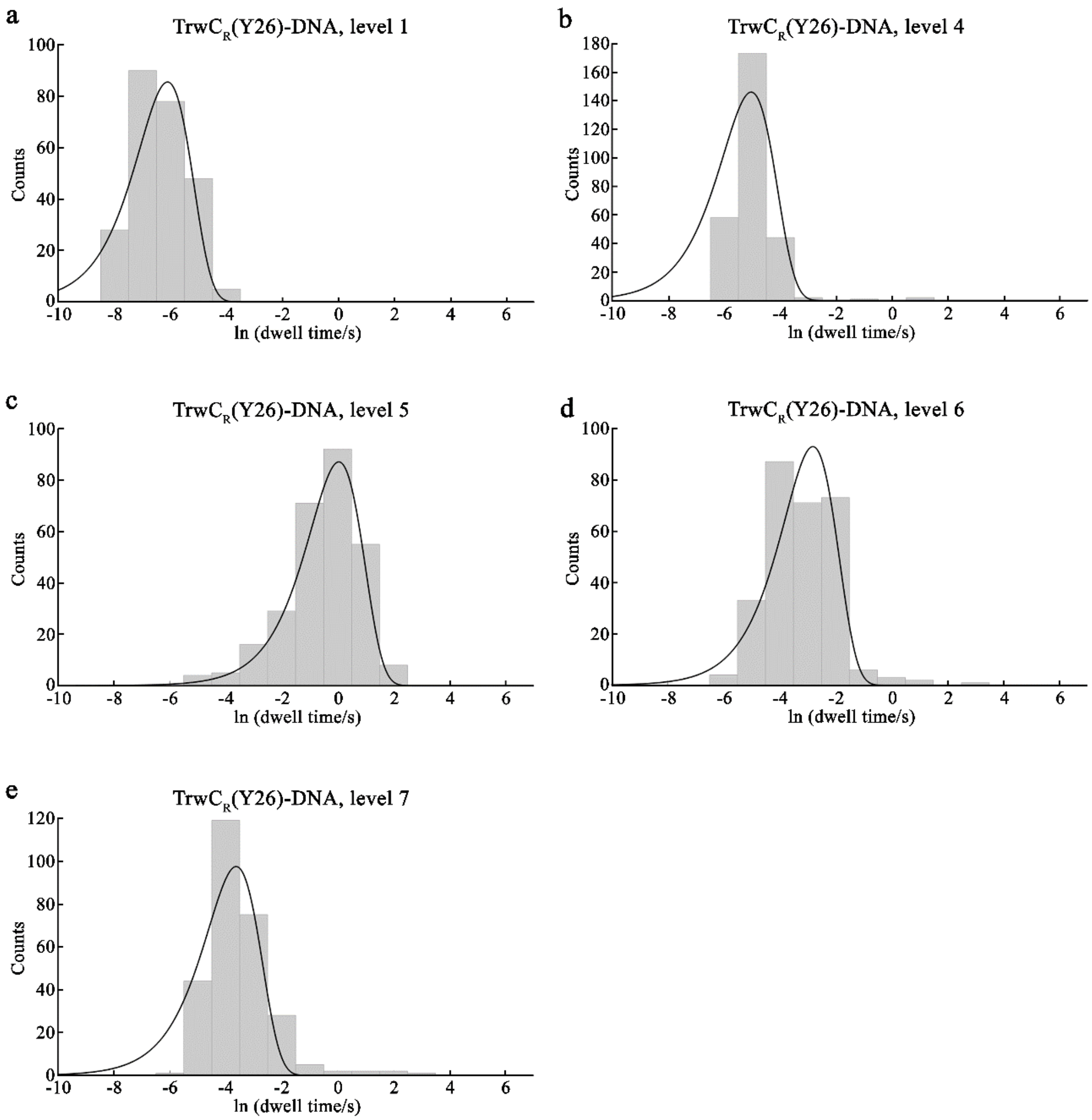

Figure S5. Dwell time distributions of a TrwCR(Y26)-DNA complex during translocation. Histograms of dwell times in levels 1, 4, 5, 6 and 7, obtained at +120 mV, are shown in panels a, b, c, d, and e, respectively. Levels 2 and 3 are shown in Figure 5 of the main text. Rate constant values are included in Table 1.

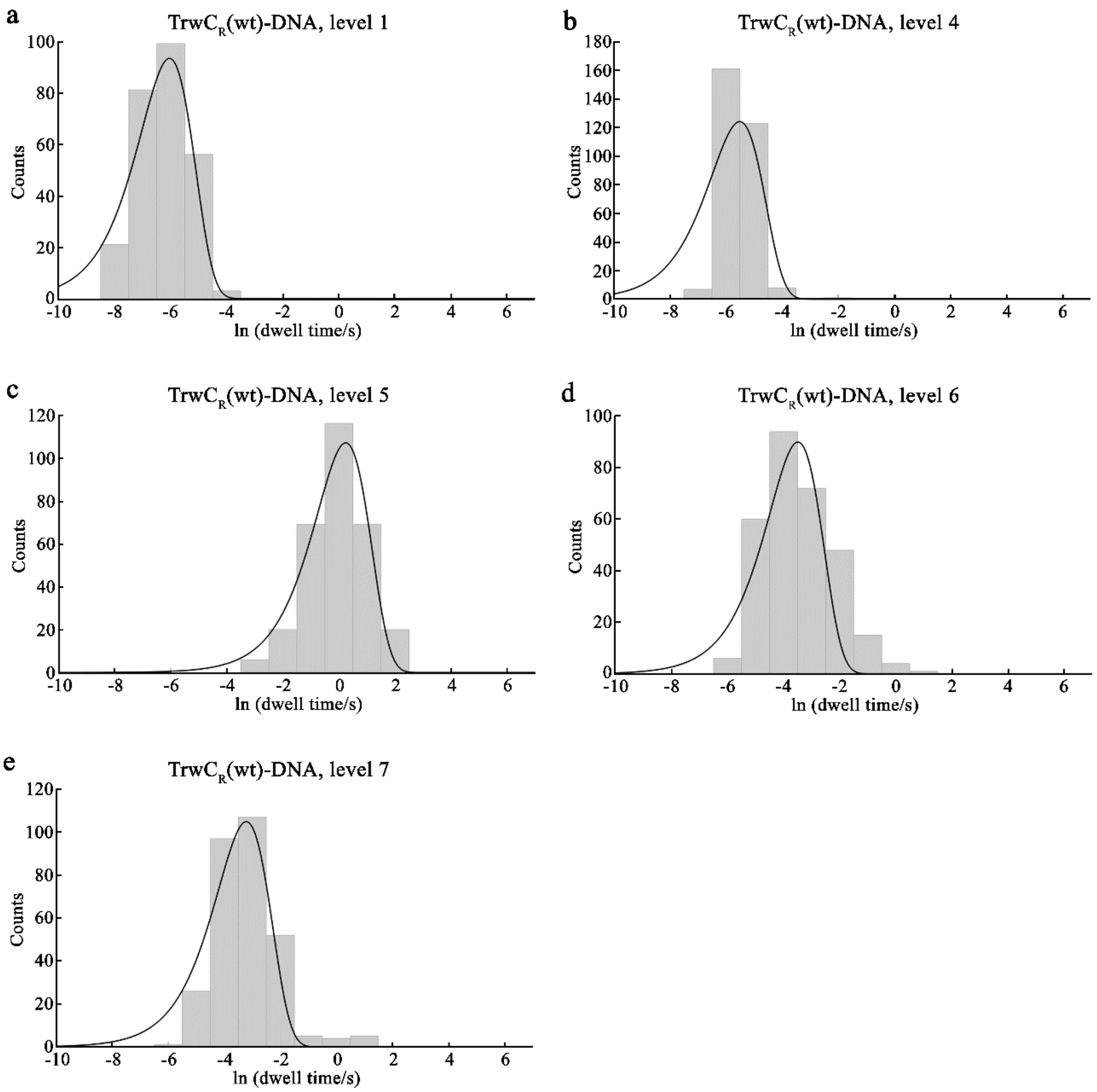

Figure S6. Dwell time distributions of a TrwC$_R$(wt)-DNA complex during translocation. Histograms of dwell times in levels 1, 4, 5, 6 and 7 obtained at +120 mV are shown in panels a, b, c, d, and e, respectively. Levels 2 and 3 are shown in Figure 5 of the main text. Rate constant values are included in Table 1.

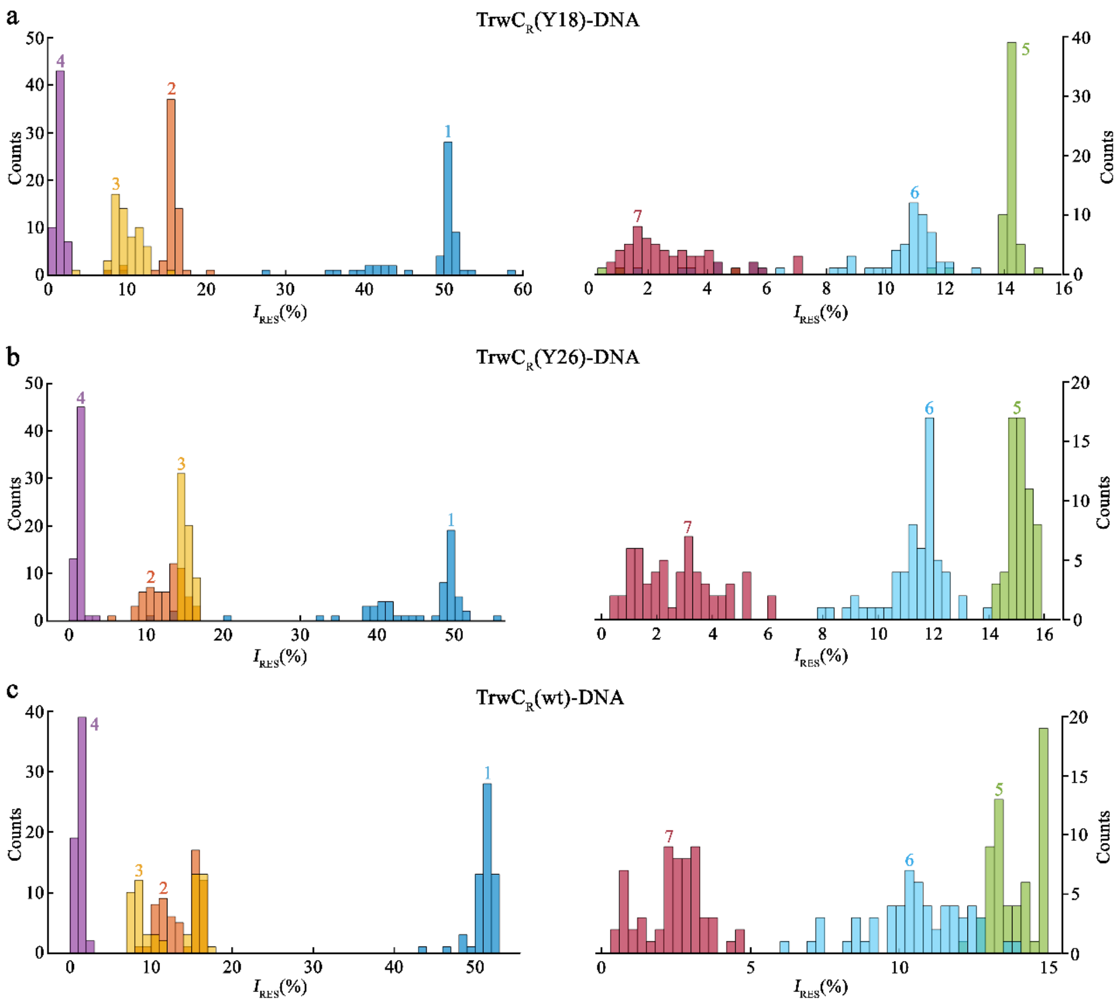

Figure S7. Histograms of the residual current levels expressed as I_{RES}(%). Event histograms of I_{RES}(%) at +120 mV observed during the co-translocational unfolding of the TrwC$_R$(Y18)-DNA complex (a), TrwC$_R$(Y26)-DNA complex (b) and TrwC$_R$(wt)-DNA complex (c). I_{RES}(%) = (I_{RES}/I_O) x 100, where I_{RES} is the current flowing during a blockade and I_O is the current through the unblocked pore. Each of the seven current levels has the same colour code in the three protein-DNA complexes.

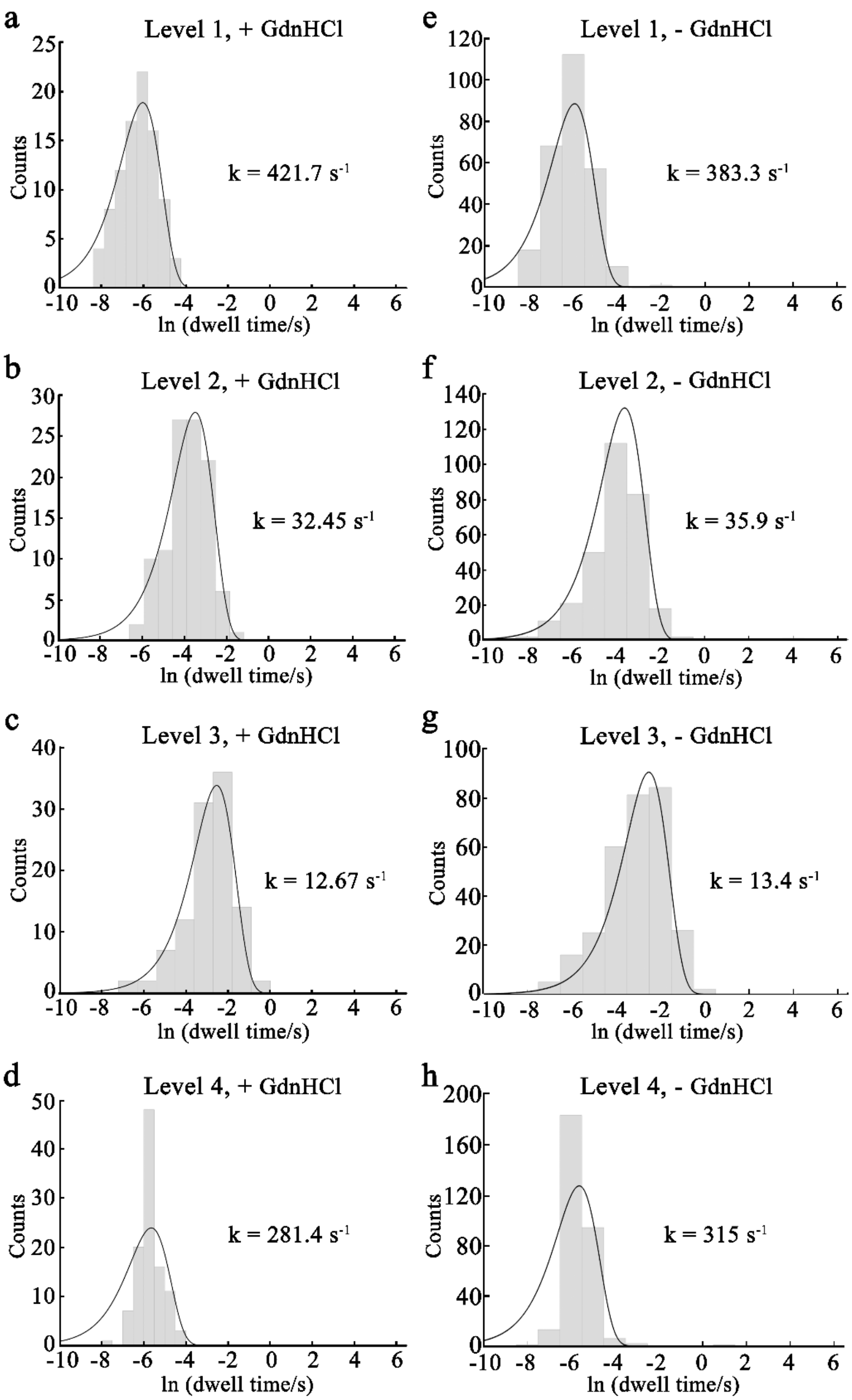

Figure S8. Dwell time distributions of a TrwC$_R$(Y18)-DNA complex during translocation in the presence and absence of a denaturant agent. Histograms of dwell times in levels 1, 2, 3 and 4, obtained at +120 mV in the presence of 0.9 M guanidinium chloride at the trans compartment, are shown in panels a, b, c and d, respectively. For comparison, panels e, f, g and h show the histograms of the same levels in the absence of the denaturant agent.

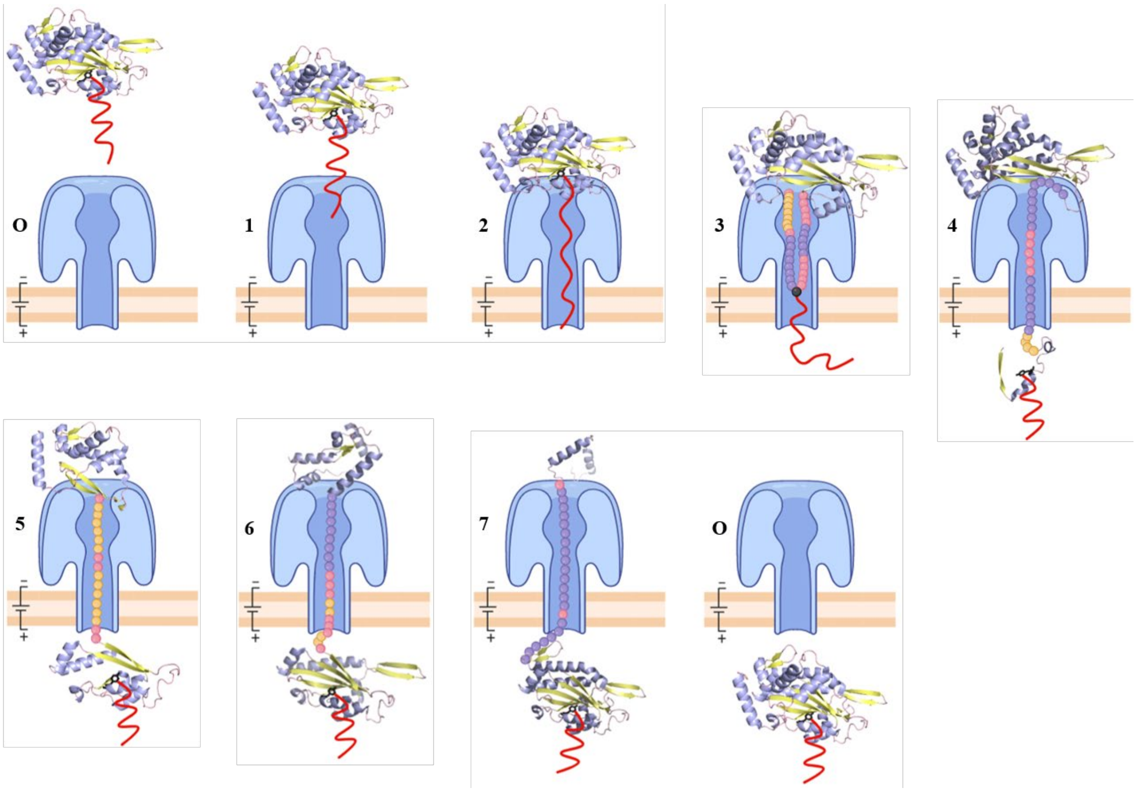

Figure S9. Molecular model for the translocation of a TrwC_R(Y18)-DNA complex through an α-HL pore. DNA is represented in red, bound to residue Y18 in TrwC_R protein (pdb 1zm5)[43]. According to the type of secondary structure, β-strands are colored in yellow, α-helices in purple and loops in pink. Level O: TrwC_R-DNA is in solution and the pore is unoccupied. An ionic current flows through the pore. Level 1: initial DNA contact with the α-HL vestibule. Level 2: DNA threads into the pore and the pulling force place TrwC_R at the top of the pore. Level 3: TrwC_R is pulled from residue Y18, so two peptide segments must enter the pore at the same time (residues 1-17 plus residues 19-33). Level 4: co-translocational unfolding of the two α-helices that connect β1 and β2 strands (residues 38-61) and a loop (residues 62-79), which precedes the central β core of the structure. Level 5: unfolding of the central core (β3-β7 strands, residues 80-168). Accordingly, this is the level that requires more time in the translocation process (Table 1). Refolding of TrwC_R protein at the trans site would act as a pulling force. Level 6: initial unfolding of the fingers domain (residues 169-onwards). Level 7: the remainder of the protein diffuses through the pore, which opens again and returns to Level O.

Table S1. Rate constants (s^{-1}) of co-translocational unfolding for TrwC$_R$(Y18)-DNA complex at different voltage values.

Step	100 mV	110 mV	120 mV	130 mV	140 mV
1 → 2	420<br>[343 – 497]	371<br>[282 – 459]	379<br>[315 – 443]	327<br>[288 – 366]	312<br>[266 – 359]
2 → 3	28<br>[25 – 30]	27<br>[24 – 30]	37<br>[35 – 39]	36<br>[33 – 39]	36<br>[31 – 42]
3 → 4	11<br>[10 – 13]	11<br>[9 – 12]	12<br>[10 – 15]	14<br>[11 – 17]	16<br>[14 – 18]
4 → 5	286<br>[149 – 424]	259<br>[125 – 393]	275<br>[161 – 389]	270<br>[104 – 437]	269<br>[120 – 418]
5 → 6	0.88<br>[0.71 – 1.04]	0.99<br>[0.79 – 1.19]	0.98<br>[0.82 – 1.13]	0.92<br>[0.79 – 1.06]	0.96<br>[0.85 – 1.07]
6 → 7	25<br>[18 – 33]	22<br>[14 – 30]	22<br>[16 – 28]	20<br>[12 – 27]	22<br>[12 – 32]
7 → O	35<br>[25 – 46]	45<br>[31 – 60]	36<br>[25 – 47]	30<br>[24 – 36]	28<br>[23 – 33]

Rate values (s^{-1}) were derived from exponential fits to dwell-time histograms, in which the data from at least three independent experiments were compiled; 95% confidence interval is provided in brackets ($n \approx 300$).

Table S2. Rate constants (s^{-1}) of co-translocational unfolding for $TrwC_R$(Y26)-DNA complex at different voltage values.

Step	100 mV	110 mV	120 mV	130 mV	140 mV
1 → 2	437<br>[351 – 522]	372<br>[260 – 484]	437<br>[315 – 559]	420<br>[308 – 531]	352<br>[266 – 438]
2 → 3	1148<br>[1111 – 1185]	1183<br>[570 – 1796]	1051<br>[985 – 1116]	1539<br>[1082 – 1997]	2006<br>[1969 – 2043]
3 → 4	0.14<br>[0.09 – 0.20]	0.12<br>[0.08 – 0.15]	0.17<br>[0.15 – 0.20]	0.19<br>[0.10 – 0.28]	0.32<br>[0.20 – 0.45]
4 → 5	158<br>[112 – 204]	155<br>[65 – 246]	155<br>[106 – 204]	158<br>[92 – 223]	167<br>[88 – 247]
5 → 6	1<br>[0.80 – 1.20]	1.15<br>[0.94 – 1.36]	0.96<br>[0.81 – 1.11]	1.13<br>[0.68 – 1.58]	0.94<br>[0.80 – 1.08]
6 → 7	16<br>[11 – 21]	14<br>[7 – 22]	18<br>[13 – 23]	21<br>[10 – 32]	18<br>[13 – 24]
7 → O	33<br>[25 – 42]	42<br>[34 – 49]	34<br>[27 – 41]	33<br>[21 – 45]	31<br>[23 – 39]

Values (s^{-1}) were derived from exponential fits to dwell-time histograms, in which the data from at least three independent experiments were compiled; 95% confidence interval is provided in brackets (n ≈ 300).
